# Margarine

**Published:** 1919-05-31

**Authors:** 


### Margarine.

THE shortage of butter during the war led to a great increase in the production and consumption of margarine. In January, 1913, 1,611 tons were produced weekly in England. In 1915 the production had risen to 3,564 tons, and in 1918 to 5,500 tons. Nor can there be much doubt that if it can be produced cheaply it will continue to be largely consumed, and it is just as well that its nutritive value and nutritive deficiency should be understood. During the war various writers commended it as a fat food superior even to butter. That it is an excellent and nourishing fat food is certain; but it is not so palatable as butter, and it is questionable whether as a food its caloric value is equal to butter. Even if its palatability and caloric value were equal to those of butter, it would still be inferior as a staple article of food, for it does not contain the fat-soluble vitamine which butter contains.

Recent researches by Professor F. Gowland Hopkins have shown that the fat-soluble vitamine in butter is as important as the water soluble vitamine present in unpolished rice, meat, milk, wheat, and eggs, and indeed there seems good reason to believe that deficiency of the fat-soluble vitamine is one of the causes, if not the chief cause, of rickets. To a certain extent, the deficiency may be made up by the fat-soluble vitamines in animal fats, but butter plays such a large part in the ordinary dietary, and contains such a large proportion of the vitamine, that the substitution of margarine for butter must mean a great diminution in the total amount of fat-soluble vitamine ingested—a diminution probably large enough to impair the health of either child or adult. If, then, margarine be used instead of butter, it will be well to add to the diet—especially in the case of children—some other substance, such as cod-liver oil, which is rich in fat-soluble vitamine. It is not unlikely that in time manufacturers of margarine will themselves add to the margarine some fat-soluble vitamine, but meantime it is just as well to point out the deficiency of margarine in this important respect.

